# Unexpected post-operative haemorrhage: Could it be splenosis?

**DOI:** 10.1093/jscr/rjac540

**Published:** 2022-12-28

**Authors:** Andrea Boerkamp, Ashwin Das, Marie Shella De Robles

**Affiliations:** Department of Surgery, Shoalhaven District Memorial Hospital, Nowra, New South Wales, Australia; Graduate School of Medicine, University of Wollongong, Keiraville, New South Wales, Australia; Department of Anatomical Pathology, Wollongong Hospital, Wollongong, New South Wales, Australia; Department of Surgery, Shoalhaven District Memorial Hospital, Nowra, New South Wales, Australia; Graduate School of Medicine, University of Wollongong, Keiraville, New South Wales, Australia; Department of Surgery, Wollongong Hospital, Wollongong, New South Wales, Australia

## Abstract

Ectopic splenic tissue can be classified as accessory spleen, polysplenia or a phenomenon termed as splenosis. Once believed a rare occurrence, the incidence of splenosis is now thought to be significantly higher. Generally, splenosis is asymptomatic and discovered incidentally during operation, imaging or at autopsy. The case presented herein describes an incidental finding of an intraabdominal splenosis, which was subsequently biopsied to investigate for peritoneal metastatic disease. The biopsied tissue subsequently caused significant post-operative haemorrhage. Past medical history and specific pre-operative imaging modalities for patients presenting with asymptomatic peritoneal or intra-abdominal nodules are discussed. Splenosis is highlighted as a common condition to consider prior to invasive investigations.

## INTRODUCTION

Splenosis is the autotransplantation of splenic tissue in one or more ectopic locations due to splenic injury and is most usually caused by traumatic rupture or surgical instrumentation. Splenosis was previously believed to be a rare occurrence; however, more recent studies have indicated a higher incidence than once thought. Intraabdominal splenosis is typically asymptomatic; however, it can also be associated with abdominal pain, intestinal obstruction, haemorrhage and hydronephrosis. The case presented here exemplifies an important consideration for surgeons and radiologists when peritoneal deposits are demonstrated on computerized tomography (CT) imaging in conjunction with a history of prior splenectomy or splenic injury.

## CASE REPORT

**Figure 1:**
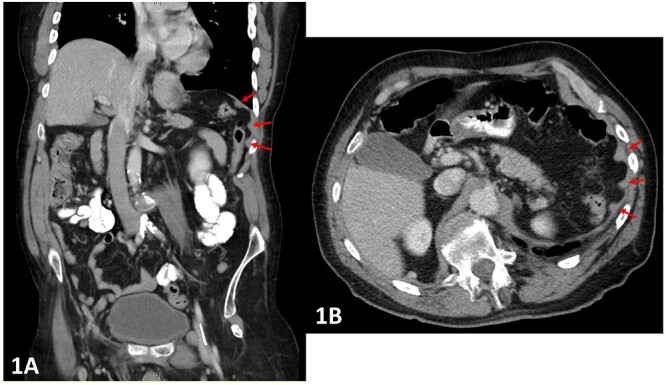
(**A** and **B**) Pre-operative CT abdomen (A—coronal, B—transverse planes) with multiple peritoneal nodules indicated by the arrows.

**Figure 2:**
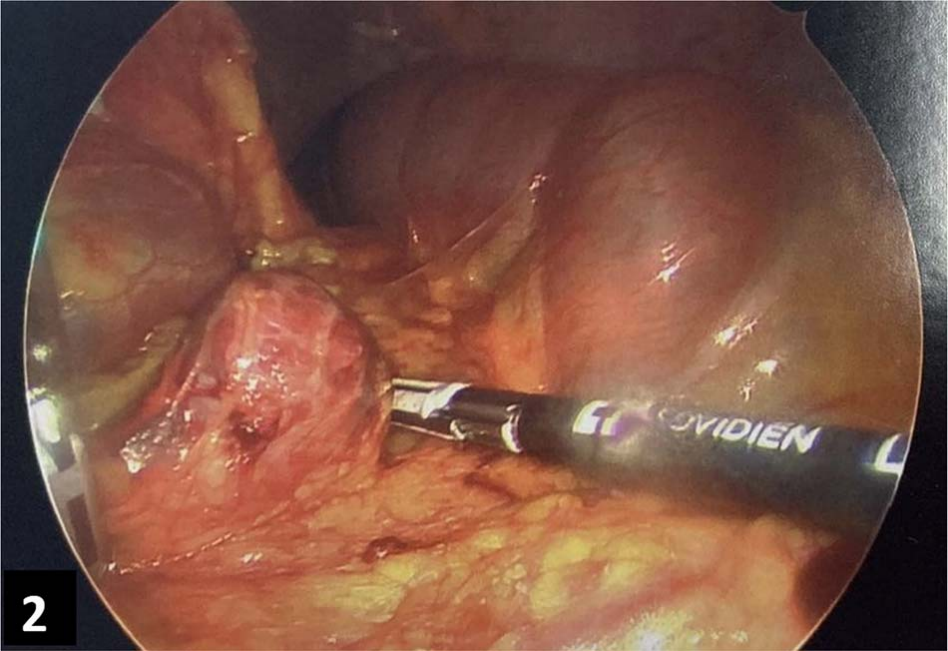
Intra-operative imaging of bleeding omental splenosis nodule.

**Figure 3:**
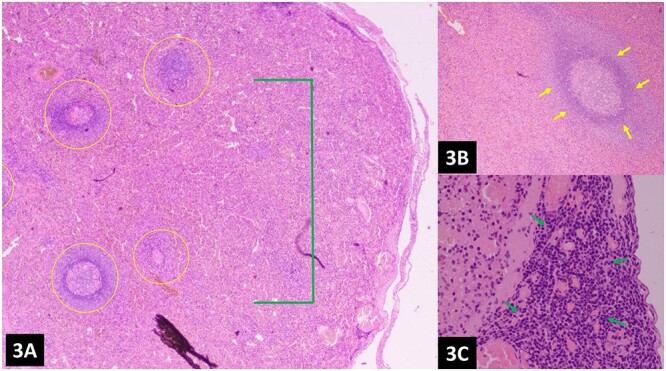
(**A**–**C**) Histopathology of the omental nodules reveals normal splenic tissue; the spleen can be recognized by red pulp (network of splenic cords and sinusoids filled with blood) and white pulp (lymphoid tissue usually surrounding splenic blood vessels), indicated by green and yellow arrows, respectively.

A 64-year-old male underwent an elective colostomy formation for obstructive defecation. The patient had a background of T12 spinal cord injury and resultant incomplete paraplegia from a motor vehicle accident 34 years prior. The same accident caused a traumatic splenic injury, resulting in splenectomy. A pre-operative CT abdomen and pelvis was completed before the elective procedure. This revealed multiple hyperdense peritoneal deposits ranging from 1 to 3 cm (Fig. 1A and B), and a biopsy was recommended. Intraoperatively, several deposits ranging from 1 to 3 cm were observed throughout the greater omentum, and biopsies were obtained for histological analysis. No intraoperative complications were encountered. However, the patient had intraabdominal bleeding on the first post-operative day and required a return to the theatre. Exploratory laparoscopy revealed multiple bleeding nodules in the omentum (Fig. 2) and a subsequent partial omentectomy was performed. There was no other source of intraabdominal bleeding on thorough re-inspection. Histopathology of the nodules was consistent with splenic tissue (Fig. 3A–C), confirming the suspicion of intraabdominal splenosis mimicking peritoneal carcinomatosis pre-operatively and causing the intraabdominal bleeding post-operatively. The patient developed a pulmonary embolism and required anticoagulation a week later. He recovered well from the operation and complicating pulmonary embolism, but he needed a short admission to a rehabilitation unit for physical conditioning. On follow-up, he is recovered and managing his colostomy well.

## DISCUSSION

Splenosis is an acquired condition from splenic trauma, which initiates the seeding of splenic tissue throughout neighbouring body cavities. The pathogenesis involves splenic cells implanting into a receptive tissue bed where the local blood supply is recruited, promoting growth into partially functional splenic tissue [2]. Nodules can be solitary or numerous but generally remain <3 cm, owing to the limitation in blood supply from the surrounding tissue. Seeded splenic tissue can implant in multiple locations, including regions of the abdomen, pelvis and thorax. Rare locations include the liver, pancreas and kidneys. Other rare locations are the brain and subcutaneous tissue, which supports the concept that splenosis may also occur due to haematogenous spread [3].

Splenosis develops between 5 months and 32 years following splenic injury, with a mean time to development of 10 years [4]. Once thought to be a rare condition, it is believed that splenosis may affect as many as 65% of patients with a history of splenic trauma [5]. Splenosis is generally asymptomatic and incidentally found during unrelated surgery or imaging, but rarely, may present with symptoms of abdominal pain, bowel obstruction or haematological disease. Splenosis is embryologically distinct from the other forms of ectopic splenic tissue (accessory spleen and polysplenia) as splenosis does not have a blood supply arising from the splenic artery [6].

Splenosis has been described as being histologically distinct from the normal splenic tissue, accessory spleen and polysplenia; however, literature suggesting the architecture may be identical also exists [7]. Morphological differences between accessory splenic tissue and splenosis are displayed in Table 1. Splenosis may mimic other intraabdominal pathologies, particularly metastatic deposits along the hepatic margin, in the pancreas, in mesenteries, over surfaces of the colon and in intrathoracic lesions [8]. History of splenic trauma or splenectomy is an important consideration when planning a further investigation of such findings. It has been shown that CT imaging alone is not sensitive enough to distinguish splenosis, particularly from metastatic deposits [3]. Imaging modalities which can confirm splenic tissue include a Technetium (Tc-99 m) sulphur colloid scan, which will demonstrate increased uptake in ectopic splenic tissue of at least 2 cm in diameter. If the Tc-99 m sulphur colloid does not confirm splenic tissue, a Tc-99 m-tagged heat-damaged red blood cell scan with autologous erythrocytes remains the gold standard of imaging because it can specifically confirm the splenic tissue [3,8]. Targeted imaging of patients with a history of splenectomy or splenic trauma may prove useful in cases of possible splenosis, as unnecessary surgery may be avoided.

**Table 1:** Morphological differences between splenosis and accessory spleens [1]

	Splenosis	Accessory spleen
Location	Any intra- and/or extra-peritoneal location	Usually confined to splenopancreatic or gastrosplenic ligaments
Number	As many as 400	<6
Blood supply	Surrounding tissue	Splenic artery
Histology	Varied—lacks trabecular structure, less elastic tissue	Uniform, normal
Shape	Varied, sessile or pedunculated, lack hilum	Rounded, may be shaped similar to normal spleen, has a hilum
Size	<3 cm, limited by surrounding blood supply	<1 cm but can be of any size and will hypertrophy due to splenectomy

The case discussed here presents important considerations when planning for further investigation of patients presenting with incidental findings of asymptomatic peritoneal nodules and a background of splenic trauma and subsequent splenectomy. In such cases, the use of the suggested imaging modalities can assist clinicians to determine the origin and nature of these lesions, thereby avoiding unnecessary surgical intervention.
